# Puerperal Anasarca

**Published:** 1866-06

**Authors:** J. P. Ross

**Affiliations:** Physician to the Cook County Hospital


*Puerperal Anasarca.* By J. P. Ross, M. D., Physician to the Cook County Hospital.

(Read before the Chicago Medical Society.)

On the evening of Dec. 17, 1865, I was called in great haste to visit Mrs. P——, living half a block from my residence. I hastened at once to the bedside of the patient, and found her in convulsions. The extremities on both sides were affected with clonic spasms; eyes rolled upward and outwards; stertorous breathing and moaning; a bloody frothing at the mouth; and in a state of unconsciousness. Efforts were simply used to prevent the patient from injuring herself. The paroxysm lasted from ten to fifteen minutes, when she recovered with considerable mental confusion and delirium.

On making further examination, I found the eyelids and face puffy, and the feet and legs swollen. She was in the seventh month of utero-gestation, and was the mother of one child. The nurse states that the dropsical symptoms had been observed for a fortnight, and for several days she had been very irritable, complained much of headache, and had delirium at night. The urine was scanty, with pain in passing it, and high colored. She had complained of pain in the small of the back. A homœopathic professor of midwifery had been in daily attendance, and was engaged to wait upon the patient through her lying-in. On learning the situation of affairs, I stated to the friends the grave nature of the case and the necessity for active interference, and advised them, if they did not propose to make a permanent change of physicians, to send at once for their medical attendant. This they thought best to do, and after remaining an hour, administering from time to time chloroform by inhalation, the patient got into a quiet sleep and I took my leave.

On the 1st of January, 1866—a fortnight from my first visit—I was waited upon by the husband of the patient, who stated that his wife was no better, and desired me to see her at once and take charge of the case. At this time the dropsical condition had very much increased, the serous cavities contained a quantity of fluid—especially the peritoneal;—the gravid uterus could be floated from side to side and from before backwards, and made only a smaller portion of the contents of the abdominal cavity, the larger portion consisting in effused fluid. Headache, delirium and convulsive twitchings occurred every night, to overcome which the patient had used by inhalation large quantities of chloroform in addition to infinitesimals. The pulsations of the fœtal heart were distinctly heard. The urine was scanty, and on boiling it a large coagulum was formed, which did not dissolve by the addition of nitric acid. The condition ot the patient was deplorable, and had steadily grown worse. It became a serious question to me if the chances of recovery for the patient would not be promoted by the induction of premature delivery. A consultation was asked for.

Jan. 2d. Dr. Byford in council. After a careful investigation of the case, Dr. B. concluded, 1st, That the pressure of the gravid uterus upon the kidneys was not the cause of the renal affection; 2d, That this disease occurred independent of the state of pregnancy; and, 3d, That the chances of recovery from it would not be greatly promoted by delivery. It was decided to give active treatment a trial in removing the uræmia and renal congestion. Ordered 10 grs. each of calomel and jalap, to be followed in six hours with a bottle of the solution of the citrate of magnesia.

Jan. 3d. 9 o'clock A. M. Cathartic operated thoroughly; urine scanty. Ordered 10 grs. of acet. potash and 10 minims of the wine of colchicum seeds every four hours, and also sol. cit. magnes. in sufficient quantity to keep up a slight drain from the bowels. In the evening the patient complained of pain at the top of the head; was restless and feverish. Ordered 10 grs. Dover powder at once, and a vapor bath: which was directed for the occasion by placing a lighted alcohol lamp under a hard-bottomed chair, on which the patient was to be seated—*in puris naturalibus*—and surrounded with blankets to confine the vapor around the body; and to remain there as long as the strength of the patient would hold out.

Jan. 4th. Patient rested well; says she "feels better than she has in a month, but too weak to raise a finger." Œdema seems less; occasional slight uterine contractions; urine still scanty and albuminous; continue the colchicum and potash. In the evening, ordered the vapor bath repeated, notwithstanding the patient protested that she was too weak, and it would surely bring on labor.

Jan. 5th. 9 o'clock A. M. Labor pains had continued since the bath regularly every fifteen or twenty minutes. On examination, found liquor amnii evacuated; presentation natural; and the os uteri soft and yielding, and dilated to a diameter of two or two and a half inches. At 11 o'clock A. M., she was delivered of a living child, which had reached about eight months of fœtal life.

During the whole period of labor no indications of convulsions were manifested, yet chloroform was used during the larger portion of the time during the existence of the pains, and six ounces were used up. The usual services were rendered after delivery, and the patient was left comfortable. No medicine was prescribed.

Jan. 6th. Patient feels well; urine passed three times during the night, very copiously. No medicine.

Jan. 7th. Bowels moved several times, watery discharges; urine free; dropsical effusion very much less; mammary secretion established; pulse 80, soft; complains of exhaustion. Ordered tr. ferri mur., gtts. xii., ter. die., and good nutriment.

Jan. 9th. Dropsy almost entirely gone; urine contains but a trace of albumen; functions normal; complains of weakness. Ordered quiniæ, gr. ii, ferri sul., gr. i., ext. nux vomica, gr. ss., in pil. ter. die. and porter.

Jan. 18th. Dressed and about the house; recovery complete. Infant doing well.

*Remarks.*—The above case is interesting, in the first place, as showing the results of treatment. For the first fortnight, the patient was under trial of homœopathic remedies. That these were perfectly inert is evident from the disease making steady progress until it culminated in convulsions. During the following fortnight the treatment was the same (which was nil) with the addition of chloroform by inhalation. This remedy seemed to render the nervous system more tolerant of the uræmic poison, but did not aid, in any way, in getting rid of it, or in removing that state of the kidneys which produced the toxæmia and dropsy. On the contrary, the disease continued to grow worse. Then a moderately heroic treatment was tried, which consisted in remedies to act thoroughly on the bowels, kidneys and skin. These were followed by results which we believe were produced by the treatment, and were, 1st, premature delivery, which progressed to a successful termination, not a symptom of convulsions showing itself; 2dly, the renal congestion gave way rapidly, which was manifested by a free secretion of urine and a disappearance of albumen; and, 3dly, the intestinal mucous membrane coming to the aid of the kidneys; the two relieved the system of dropsy, and the patient most rapidly recovered from a dangerous and alarming condition.

The independence of the morbus Brightii of the state of pregnancy in this and similar cases may be fairly questioned. Keeping in mind the length of time which the disease had lasted, and the results produced, mark how rapidly recovery occurred after delivery, and mark another fact, that no permanent improvement occurred in the quality or quantity of urine until delivery was effected. In these particulars this case does not stand alone, for both facts were patent in all the cases I have treated for several years.

In what way utero-gestation produces this morbid condition of the kidneys may not be known. The theory of pressure of the gravid uterus upon the renal veins could be scarcely adopted in this case, for the walls of the abdomen were not tense, nor the uterus confined in any way, but seemed to float—frequently changing its position—in the fluid of the peritoneal cavity.

*Question.*—What is the cause (or what are the causes) of the complication of Bright's disease with utero-gestation?

